# Testing the Dose–Response Specification in Epidemiology: Public Health and Policy Consequences for Lead

**DOI:** 10.1289/ehp.7691

**Published:** 2005-05-10

**Authors:** Stephen J. Rothenberg, Jesse C. Rothenberg

**Affiliations:** 1 National Institute of Public Health, Center for Research in Population Health, Cuernavaca, Morelos, Mexico; 2 University of Sydney, Faculty of Economics and Business, School of Economics and Political Science, Camperdown, New South Wales, Australia

**Keywords:** child IQ, dose response, health benefit, health policy, lead

## Abstract

Statistical evaluation of the dose–response function in lead epidemiology is rarely attempted. Economic evaluation of health benefits of lead reduction usually assumes a linear dose–response function, regardless of the outcome measure used. We reanalyzed a previously published study, an international pooled data set combining data from seven prospective lead studies examining contemporaneous blood lead effect on IQ (intelligence quotient) of 7-year-old children (*n* = 1,333). We constructed alternative linear multiple regression models with linear blood lead terms (linear–linear dose response) and natural-log–transformed blood lead terms (log-linear dose response). We tested the two lead specifications for nonlinearity in the models, compared the two lead specifications for significantly better fit to the data, and examined the effects of possible residual confounding on the functional form of the dose–response relationship. We found that a log-linear lead–IQ relationship was a significantly better fit than was a linear–linear relationship for IQ (*p* = 0.009), with little evidence of residual confounding of included model variables. We substituted the log-linear lead–IQ effect in a previously published health benefits model and found that the economic savings due to U.S. population lead decrease between 1976 and 1999 (from 17.1 μg/dL to 2.0 μg/dL) was 2.2 times ($319 billion) that calculated using a linear–linear dose–response function ($149 billion). The Centers for Disease Control and Prevention action limit of 10 μg/dL for children fails to protect against most damage and economic cost attributable to lead exposure.

Few researchers doubt that lead exposure has significant health consequences at levels below those considered medically acceptable just decades ago, although there is still debate over what levels of lead exposure, if any, can be considered harmless. Key to this debate is determining the form of the dose–response function describing how the amount of exposure is related to the magnitude of the health effect.

There are two basic forms of the dose–response function for lead: a simple linear model, in which the increase in health effect is a linear function of increasing blood lead concentration (BPb), and a nonlinear model, in which the amount of health effect change attributable to lead changes according the region of the dose–response curve studied. A special case of the nonlinear dose–response function is a threshold model in which the response to lead decreases as a function of decreasing dose until it reaches a lead dose below which there is no further detectable change in health. An alternative threshold model is one in which the response to lead changes as a function of increasing dose until an upper lead bound is reached, at which point the increase in health damage exceeds predictions, as in cases of high doses producing organ damage.

Although epidemiologists have become increasingly sophisticated in construction and diagnosis of models describing their data, as a whole, we generally pay much less attention to systematically and rigorously addressing the specification of the dose–response function. A number of public health issues depend on adequately specifying the form of the dose–response function for lead, chief among them regulatory action.

Cost–benefit analyses should form the backbone of regulatory decisions regarding permissible exposures or background concentrations of toxic substances in both population and occupational settings. In such an ideal world, the savings in health care, disability, and productivity gain realized from reducing exposure to a particular substance are compared to the cost required to achieve that reduction in exposure. Policy analysts seek the “sweet spot,” where the marginal costs of lead reduction equal the marginal benefits (i.e., where the slopes of the cost function and benefits function are equal) (Pacala et al. 2003). Even if in the real world less easily quantifiable factors affect regulatory decisions, all parties to regulation have some notion of costs and benefits in mind when presenting their cases to regulatory agencies.

One recent publication (Grosse et al. 2002) presented data on the economic benefits of nationwide lead reduction due to childhood IQ (intelligence quotient) loss attributable to lead over the last 25 years. These authors conservatively used a linear dose–response function of lead–IQ as part of their model, stating that there was insufficient evidence to determine the shape of the dose–response function. The economic savings predicted by their model were in the range of hundreds of billions of dollars over the lifetime of a yearly birth cohort.

The lead–health dose–response function selected for the benefits model has clear implications for policy decisions based on it. A threshold model suggests that once reductions of population level of lead reach the threshold, further lowering of lead would have no beneficial health or economic consequences. The current Centers for Disease Control and Prevention (CDC) action limit of > 10 μg/dL for children (CDC 1991) would be justifiable on health grounds alone if there were a threshold somewhere near that limit. A linear model suggests that equal reduction in population BPb is accompanied by equal reduction in health consequence from any starting level of lead. Under a linear dose–response model, even though the health benefit would continue to increase with further population lead reduction, the present CDC action limit might be justifiable on economic grounds if the cost of further population BPb reduction far exceeded the recoverable economic benefits. A nonlinear model, especially one in which health benefits are greater for lead reduction nearer the population’s zero lead point than farther from it, would argue for further reduction in population lead levels and CDC action limits if the accelerated health benefit at lower lead levels exceeded the increased costs of lead reduction to those levels.

In this article, we present a critical examination of the dose–response function in a widely studied area of epidemiologic research with lead: childhood IQ. We present easily accessible statistical techniques useful for deciding among alternative dose–response functions and for testing whether residual confounding resulting from possible misspecification of model control variables affects the dose–response function. We apply our dose–response modeling results to the benefit model noted above to calculate changes in economic benefits realized from using a statistically adequate dose–response function. The results are placed in the context of public health policy and regulation.

## Materials and Methods

### Data sets.

Eight prospective studies of lead exposure have used child IQ or developmental index as the outcome measure, with outcome measured at least to 5 years of age, of which seven (Baghurst et al. 1992; Bellinger et al. 1992; Canfield et al. 2003; Dietrich et al. 1993; Ernhart et al. 1989; Schnaas et al. 2000; Wasserman et al. 1997) agreed to participate in a pooled analysis study; combining the data sets produced a study sample of 1,333 with a 0.1–71.7 μg/dL range of lead exposure (Lanphear et al. 2005). All studies producing data for the pooled analysis were approved by an appropriate institutional review board. Child IQ as measured by one of several versions of the Wechsler Intelligence Scales for Children (Wechsler 1967, 1974, 1981, 1991) around 7 years of age was regressed on different indices of BPb (child BPb from 6 to 24 months, peak BPb during the first 7 postnatal years, average BPb over the same time, and contemporary BPb) in multiple regression models controlling for maternal IQ and education, quality of the home environment and child–caretaker interaction [Home Observation for Measurement of the Environment (HOME); Caldwell and Bradley 1984], birth weight, and study site. Other control and confounding variables, such as child’s sex, tobacco exposure during pregnancy, alcohol use during pregnancy, maternal age at delivery, marital status, and birth order, had no significant effect in the models, did not significantly alter the IQ–lead relationship, and were not included in the final models. All lead variables in the models were natural-log transformed. All lead variables had highly significant effects on IQ (*p* < 0.0005) in the models.

We selected BPb measured contemporaneously with IQ for further analysis using the pooled data set [adjusted estimate of natural-log lead (95% confidence interval; CI) on IQ = −2.70 (−3.74 to −1.66)], because this was the measure to which Lanphear et al. (2005) devoted most attention, even though it had the second smallest coefficient among the four measures presented.

### Statistical analyses.

#### Multiple regression modeling.

The IQ data set was analyzed with the original model specifications, including log-transformed BPb, using multiple regression analyses (STATA, version 8.2; Stata Corp., College Station, TX, USA). The IQ multiple regression model was also respecified with a linear lead term.

#### Specification tests for the functional form of the lead variable (dose–response function).

The omitted variable test, or regression specification error test (Ramsey 1969), statistically tests change in model fit when any polynomial transformation of the variable in question is used in place of the original functional form of the variable. To test whether the polynomial form is superior to the original form of the variable, a chi-squared test is constructed by using the difference in two models’ chi-squared value (or the difference in two times the log likelihood of the two models), with the number of degrees of freedom determined by the number of additional variables added to the polynomial model. This is a maximum likelihood evaluation of changes in model fit and is a test of nested models, because the original specification is nested within the polynomial specification. Its principal disadvantage is that it only tests whether a polynomial specification is better than a simpler specification and does not allow direct comparison of two non-nested models each with a different specification, such as linear and logarithmic. On the other hand, it is easy to do even in the absence of “canned” statistical routines and quickly indicates whether the original variable specification can be improved by adding polynomial terms.

An accessible approach for comparing variable specification between two non-nested models is the *J*-test (Davidson and MacKinnon 1981). It can be realized by first obtaining predicted values for two models, each with a different specification of the same independent variable, and then adding the prediction of the first model to the specification of the second model and vice versa (Appendix). A clear indication in favor of one or the other specification would occur when one of these prediction-added models results in a significant value for one specification of the variable and the other prediction-added model results in a nonsignificant value. A disadvantage of this test is its low power to detect a significant improvement in variable specification. Hundreds or thousands of observations might be needed if the difference between two alternative variable specifications is subtle or the variable is measured over a limited range. Low power and limited range are not limiting factors in the present study (*n* = 1,333; BPb range, 0.1–71.7 μg/dL).

#### Testing for residual confounding.

When control or confounding variables either are omitted or their functional form is mis-specified, the resulting residuals in the model could cause an alteration in the apparent functional form of the dose–response relationship (Becher 1992). In the case of IQ, not accounting for variables such as the number of other family members, family socioeconomic status, birth or childhood trauma or serious illness, IQ of the father, or other variables that might control subject IQ could alter the measured form of the dose–response relationship for lead. If these variables are not accounted for in the experimental design by becoming part of the inclusion/exclusion criteria or they are not tested for and, where appropriate, included in the models, they may contribute to residual confounding of the dose–response curve.

Another potential cause of residual confounding occurs when the functional form of included control or confounding variables is not correctly specified. Because much statistical modeling in epidemiology is performed using some variant of generalized linear models (we used least-squares regression), modelers may assume that the linear specification of these other variables is correct. For instance, a truly nonlinear relationship between maternal and child IQ that is mistakenly modeled as a linear relationship, significant or not, will alter the residuals of child IQ over different parts of the maternal–child IQ relationship. Because the dose–response curve for lead–IQ is based on those residuals, this confounding can modify the modeled dose–response relationship.

When residual confounding is caused by a variable omitted from the design, there is little remedy available except to redesign the study and collect the data anew. Fortunately, we can account for residual confounding when it is due to misspecification of included variables. Generalized additive models (GAM) (Hastie and Tibshirani 1990) can use smoothing spline functions, among other smoothers, to fit continuous and ordinal independent variables to the dependent variable instead of predetermined linear fits as with linear regression models. Depending on the number of degrees of freedom allotted to the splines, the technique can follow complex nonlinearity in the relationship between independent variables and the dependent variable, nonlinearity that might be difficult to account for by parametric functions. The penalty for increasing the complexity of the spline fit is the use of more degrees of freedom in the model. GAM yields no parameters readily summarizing the relationship between independent variables and the dependent variable. There is no disadvantage, however, if we want to use GAM to characterize the possibly complex relationships among independent control variables and the dependent variable to avoid having incorrect residuals affect the parametric dose–response relationship, as has been previously shown with simulations (Benedetti and Abrahamowicz 2004).

GAM allows calculation of the gain from the spline fit over a linear fit by assessing the increase in deviance of the fit of the linear characterization of the variable compared with the spline fit characterization. Under the null hypothesis that nonlinearity of the smoothed function is an artifact, the gain is approximately a chi-squared distribution. Thus, approximate probability values can be calculated for improvement of fit using the spline function. A significant gain indicates that the original linear or any other specification of the variable was a poorer fit to the data than is the spline fit. The procedure also gives a total model gain and model gain significance value.

We used cubic-spline GAM modeling of IQ. We modeled all continuous and ordinal variables with cubic splines with 2, 3, and 4 degrees of freedom. We constructed three alternative models based on the basic model above. In the first series of GAM models, we used untransformed BPb (linear BPb) spline modeled with the same number of degrees of freedom as the control variables. A significant gain in the spline-modeled untransformed BPb term would indicate that the original linear BPb specification could be improved upon, after correcting for any nonlinearity in the control variables.

In the second series of GAM models, we substituted the natural-log–transformed BPb variable for the linear BPb variable of the first model, allowing the number of degrees of freedom of the spline fit to vary as in the first model. An insignificant gain of the natural-log–transformed lead variable would indicate there was no improvement detected in the fit to the dependent variable by spline modeling of the log-transformed lead variable, correcting for possible nonlinearity in the control variables.

Finally, the third series of GAM models was constructed as above, except that the natural-log–transformed lead variable was held to 1 degree of freedom. This tested the original natural-log specification of the lead variable in a model where residual confounding from possible misspecification of the control variables was corrected. Insignificant gains in the other variables would suggest that their original specifications were adequate. The size of the lead coefficient was compared between the third series and the original multiple regression model to determine how much residual confounding of misspecified control variables affected the estimated size of the relationship between lead and the health outcome dependent variable.

All statistical procedures were carried out using MATLAB (version 6.5.1; Mathworks, Natick, MA, USA) and STATA 8.2.

#### The benefits model.

We used a previously published model (Grosse et al. 2002) of economic benefits showing expected dollar savings produced by population lead declines in the United States from 1976 through 1999 solely through increased population cognitive ability as measured by lead effects on child IQ. The model posits that the dollar gain in the affected cohort is a simple product of reduction in BPb over the period (micrograms per deciliter), the IQ–BPb slope (IQ per micrograms per deciliter), the earnings–IQ slope (%), the present value of lifetime earnings of a 2-year-old child (in year 2000 dollars), and the size of the 2-year-old cohort. Grosse et al. (2002) used linear IQ–lead slopes of 0.185–0.323 IQ points for each 1 μg/dL, calculated from published meta-analyses.

Instead of the linear IQ–lead slope, we substituted the change in IQ expected over the estimated 15.1 μg/dL decrease in population lead in the United States, calculated by assuming both a linear–linear and a log-linear lead–IQ dose–response function using the results of the pooled analysis presented above and then recalculated the cohort benefit.

## Results

### Lead and IQ.

Table 1 shows the lead coefficients of the different IQ models. Both linear and natural-log lead specifications were highly significant (Table 1). The omitted variable test using the linear lead variable showed a significant improvement in fit using the polynomial lead specification (*p* = 0.020), whereas the same test showed that a polynomial form of the log lead variable offered no improvement (*p* = 0.258).

The *J*-test showed that the log lead specification was still significant (*p* = 0.009) in a model with the prediction from the linear lead model added (Table 1). The alternative model, the linear lead model with the prediction from the log lead model resulted in an insignificant linear lead variable (data not shown). The results indicate that the log lead specification described the data significantly better than did the linear lead specification.

Tables 2–4 show the results of the GAM analyses. Presented results are limited to the 2 degrees of freedom spline fits because they usually resulted in the largest gains and lowest probability values, although results were similar for the 3 and 4 degree of freedom spline fits. Spline fit gains are shown only for ordinal and continuous variables because dichotomous variables cannot be fit by splines and they remain in the model unmodified.

In Table 2 the linear lead model is entirely fitted by splines. Note that the gains of all control variables were nonsignificant, suggesting adequate specification of these variables as linear. The gain of the linear lead variable was highly significant (*p* = 0.006), and the total gain of the model was also significant (*p* = 0.0142). These findings indicate that both the linear lead specification and the model as a whole better fit the data when splines were used than when the original variables were fit by linear regression. The results from Table 1, that the linear lead term did not adequately fit the data, was confirmed in Table 2, and the nonlinear (spline) fit of the lead variable was not due to residual confounding with included variables.

Table 3 shows the same spline-fit model as Table 2, but the natural-log lead term is substituted for the linear lead term. Once again, no control variable showed significant gain using the spline fit, the log-transformed lead variable gain was also nonsignificant (*p* = 0.230), and the model itself was not significantly improved by fitting the variables with splines (*p* = 0.163). There was no significant improvement in the log-linear lead–IQ fit by adjusting for possible departures from that specification.

In Table 4 the natural-log–transformed lead variable is allowed to maintain its original specification while the remainder of the variables are fit with splines. Comparison of the coefficient of the log lead variable in this model (β= −2.62) with the coefficient of the multiple regression model (β = −2.70; second coefficient from Table 1) further supports the result that there was no important misspecification of the control variables in the original multiple regression model and the log-linear form of the dose–response curve was not affected by residual confounding of variables included in the model.

**Figure 1:**
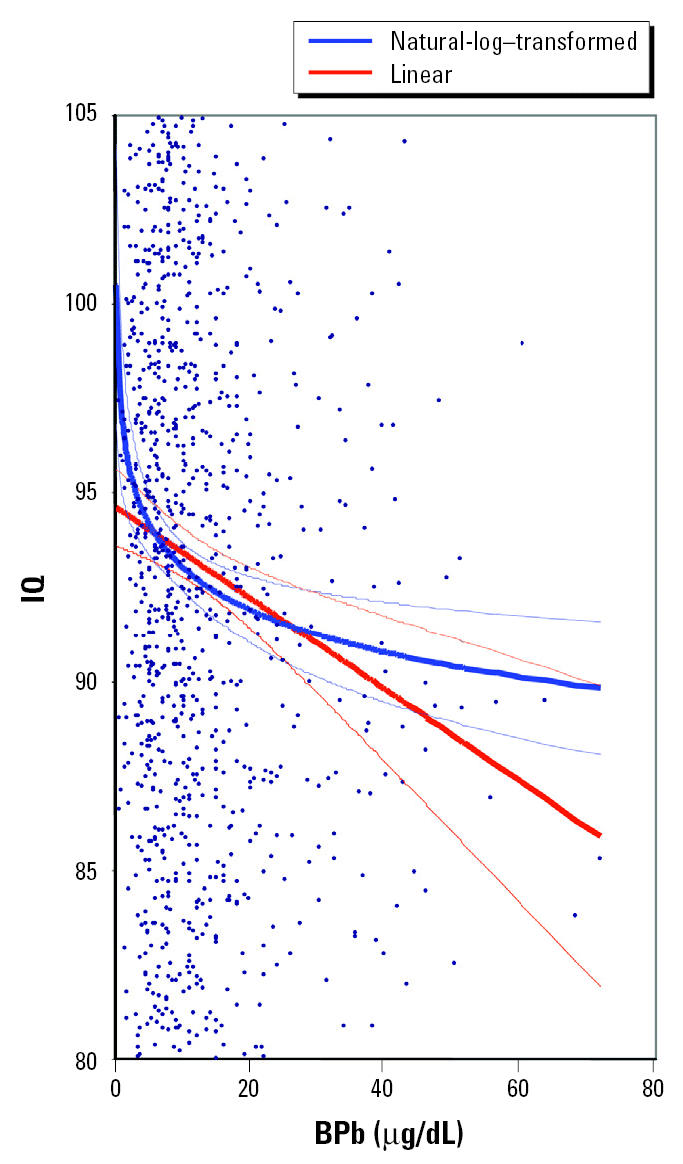
Partial regression plot of adjusted IQ (adjusted for natural-log lead model) and BPb (from Lanphear et al. 2005). The two regression lines (bold) with 95% CIs (narrow lines) represent the best-fit estimates of the relationship between IQ and BPb for natural-log–transformed BPb and linear BPb. Note that the linear BPb term overestimates the slope (change in IQ with change in BPb) of the statistically superior natural-log lead function down to 15 μg/dL and underestimates the slope < 15 μg/dL. The scatter plot does not show all data points because the *y*-axis has been expanded to show differences in regression functions.

These results strongly support the hypothesis that an adequate description of the dose–response curve for the effect of lead on child IQ is log-linear, not linear, and that residual confounding of the dose–response specification by possible misspecification of included control variables played no role. The log-linear dose–response relationship is compared with the linear dose–response relationship in Figure 1.

### Economic benefits model for lead–IQ.

Grosse et al.’s (2002) benefit model of economic gains due to lead reduction effect on IQ in the United States calculated the total year 2000 dollar savings as a result of the fall of BPb over a 23-year period. Their model postulated that the dollar benefit per cohort was benefit = *A* × *B* × *C* × *D* × *E*, where *A* is the reduction in BPb (micrograms per deciliter); *B* is the IQ–BPb slope; *C* is the earnings–IQ slope (%); *D* is the present value of earnings of a 2-year-old child (in 2000 US dollars); and *E* is the size of the 2-year-old cohort. We used their “base case” figures as follows: *C*, 2.0; *D*, $723,000; *E*, 3,800,000. In place of Grosse et al.’s *A* of 15.1 μg/dL, we used the difference in the natural-log BPb values in 1976 and 1999: BPb (1976), 17.1 μg/dL; natural-log BPb (1976), 2.84; BPb (1999), 2.0 μg/dL; natural-log BPb (1999), 0.69; difference in BPb (1976 –1999), 15.1 μg/dL; and difference in natural-log BPb (1976 – 1999), 2.15. In place of Grosse et al.’s *B* of 0.257 IQ–BPb slope (every decrease of 1 μg/dL BPb is associated with an increase of 0.257 IQ points) used in their “base case” analysis, we used the natural-log lead coefficient calculated from the pooled analysis study (Table 1, natural-log lead model), 2.70 (every natural-log unit decrease in BPb is associated with an increase of 2.70 IQ points).

The original benefits model used uncertainty in the reduction of BPb over the period studied (variable *A*) and the IQ–BPb slope (term *B*) to calculate upper and lower bounds on economic benefits. We used only the uncertainty in the IQ–BPb slope, calculated from the coefficients presented in Table 1 and from the reported meta-analysis Grosse and colleagues used (Grosse et al. 2002; Schwartz 1994) in their base case analysis. We present Grosse et al.’s original calculations based on their linear lead coefficient, the new calculations based on a log-linear dose–response relationship with 95% CIs of *B* (−3.74 to −1.66), and, for comparison, the dollar savings per cohort based on the demonstrably incorrect linear lead specification calculated from the pooled analysis study, 0.18 (95% CI, −0.26 to −0.10) (Table 1, linear lead model). These results are presented in Table 5.

Savings estimated using the correct log-linear dose–response relationship between BPb and IQ are nearly 2.2 times those estimated using a poorly fitting linear dose–response relationship for the same decrease in population BPb.

## Discussion

### Model specification.

Diagnosing model specification is an essential part of statistical modeling, particularly when ordinal and continuous variables are part of the model. Compared with the more commonly used diagnostic tests for general linear models, such as testing for distribution and homoskedasticity of residuals, formal tests of the assumed functional form of any independent variables against the dependent variable are scarcely reported in the epidemiologic literature. We often do not address functional form issues except as a by-product of adjusting residual diagnostics. For example, most lead–IQ studies in children, especially in the last 20 years, have used a natural-log–transformed lead variable to normalize the residual distribution of the model and correct for heteroskedasticity of residuals.

Although issues of the functional form of the lead–health effect relationship have occasionally been raised in the literature, notably by Schwartz (e.g., Schwartz 1994), and more recently and extensively by Grosse et al. (2002), it is common practice for the authors of applications of these studies to economic analysis to use linear approximations of the lead effect over a limited range of BPb, as did Grosse et al. (2002). Authors have not extrapolated health effects below the lower limits of lead in their data sets in the past. Data sets studying a wide range of BPb have only recently become available. Because linear and log lead specifications produce large differences in predictions only as BPb approaches zero (Figure 1), data sets including substantial numbers of very low BPb levels are required to notice, appreciate, and test for adequacy of alternative specifications. Implicit in the log-lead specification is that change in health effect with change in BPb at higher levels is small, save when lead toxicity associated with pathologic organ damage is reached.

Most studies using a log-linear dose–response relationship also neglect to comment on the public health implications of this functional form. As opposed to a linear dose–response relationship, where equal changes in health outcome are predicted for equal changes in BPb across the entire range of BPb, the log-linear relationship has the steepest slope at the lowest BPb. Health outcome changes are equal for equal proportional changes in BPb across the entire tested range of BPb. In the case of a log-linear dose–response relationship, the increase in population IQ predicted from a decrease in population BPb from 2 to 1 μg/dL is exactly the same as that predicted from a decrease in BPb from 20 to 10 μg/dL or from 40 to 20 μg/dL, although populations exposed to these different concentrations of lead will likely have different mean IQs.

We calculated the BPb change in the U.S. population between 1976 (17.1 μg/dL) and 1999 (2.0 μg/dL) used by Grosse et al. (2002) at 2.15 natural-log units change. The pooled analysis study has 38 subjects with BPb levels < 2 μg/dL. If we project the 1999 population BPb of 2.0 another 2.15 natural-log lead units down to a population lead level of 0.24 μg/dL in the indeterminate future, we can duplicate the health benefit of BPb reductions for the population already achieved by the reductions between 1976 and 1999, at least for IQ outcomes.

The log-linear dose–response function for lead, especially if it generalizes across other health outcomes, may also account for the failure of many older studies to find significant lead-related effects. In occupational studies of health effects of lead exposure, often the generally high mean BPb levels of the “exposed” groups and even the “nonexposed” control group place health comparisons among exposure levels on the flat end of the log-linear dose–response curve, where the dose–response curve approximates a nearly zero slope linear trend. Under such conditions, a very large sample size would be needed to detect significant differences among groups. If a linear model were used to specify the dose–response relationship at higher BPb, even significant effects detected in large studies would have small coefficients. The apparent “no-effect” relationship predicted by the near zero slope of a log-linear dose–response function at elevated BPb is especially notable in the occupational lead–blood pressure literature.

We do not propose that the log-linear dose–response function for BPb effect on child IQ is the “correct” dose–response function. Our analysis indicates only that it is superior to a linear–linear dose–response function. We examined two other nonlinear dose–response functions for this relationship, a third-order polynomial and a logit dose–response function. The logit function is attractive because it can model a reduction of the dose–response slope as BPb falls below currently modeled data, thus providing for the possibility of an ultralow threshold for lead effect on IQ. The polynomial function would permit modeling of a new increase of slope of the dose–response function beyond the upper limits of the data set modeled here. This would allow accounting for severe lasting effects of the pathologic changes associated with lead-induced encephalopathy. However, the alternative nonlinear dose–response functions both modeled the present data set no better than did the log-linear function, including the steeper slope at low BPb. There was a difference of < 0.2% of the variance in IQ accounted for by the lead variable among the three specifications. Because the log-linear dose–response relationship only required two parameters for complete specification and the alternatives required three parameters, we elected to use the most parsimonious specification for detailed analysis of the data set at hand.

### Public health and policy implications.

There appears to be no support for a threshold model for BPb effect on IQ. On the contrary, instead of finding a no-effect lower limit, the present study strongly suggests that most of the damage attributable to BPb occurs within the first few micrograms per deciliter of BPb within the lead range studied. Any apparent threshold will appear at the upper ranges of BPb, where the dose–response curve flattens, at least until BPb reaches the range producing frank organ damage.

In prospective lead studies of child development, including the pooled study IQ effects cited here, history of exposure is always available in the form of sequential BPb measurements of each child. We have good evidence that the log-linear lead–IQ dose–response function is not an artifact of unmeasured history of exposure and represents the best available functional description of BPb on IQ.

The drop in population lead exposure from mean BPb of 17.1 μg/dL to 2.0 μg/dL over the last quarter century produced the large health benefits calculated in Table 5. Although lead in paint and food has been specifically regulated with the goal of reducing population lead exposure, the reduction of lead in air and eventually in dust, one of the major contributors to past urban population lead exposure (Mielke et al. 1983, 1997, 1999), was by and large due to the introduction of catalytic converters for automobiles. Thus, a large part of the drop in population lead was only coincidentally achieved in response to the stated policy of reducing gaseous automobile contaminants. Fortunate though we may have been to have benefited from this accidental process, it is unlikely that further reduction in population lead exposure will be achieved without increased targeted effort.

Although many hailed the Occupational Safety and Health Administration’s 1979 regulations seeking to limit occupational exposure to 40 μg/dL (Occupational Safety and Health Administration 1979) and the CDC’s promulgation in 1991 of action limits for childhood lead exposure to 10 μg/dL (CDC 1991), it appears very likely that these limits have prevented only a small percentage of the damage associated with lead exposure. The total modeled increase in IQ from the pooled data study over the BPb range of 0.1–71.7 μg/dL was 17.7 IQ points. The improvement in IQ predicted by the log-linear model down to the CDC action limit for children was 5.3 IQ points; the remainder of the IQ improvement (12.4 IQ points) was found below the CDC action limit. If we continue to permit children and, by extension, pregnant women to maintain up to 10 μg/dL BPb without aggressive intervention to lower exposure, we are still allowing most of the preventable subclinical damage to occur.

Economic benefits realized by lead exposure reduction under a log-linear dose–response function are more than twice that previously estimated using a linear dose–response function. Updated cost studies of further population and occupational exposure reduction are long overdue. Using updated cost and benefit models, epidemiologists and health economists can determine how much additional exposure reduction is economically warranted.

In this article we only address the form of the dose–response function for lead effect on child IQ. Another well-studied area of health effects of lead exposure is the effect of lead on adult hypertension and blood pressure. A recent meta-analysis of studies examining the effect of contemporary BPb on adult blood pressure (Nawrot et al. 2002) used a log-linear dose–response relationship and found a signifi-cant effect. For every doubling of BPb, Nawrot et al. (2002) calculated a 1.0 mm Hg increase in systolic blood pressure and a 0.6 mm Hg increase in diastolic blood pressure. The pattern of increased blood pressure with increase in BPb was exactly the same as the decrease in child IQ with increased BPb. The greatest changes in predicted blood pressure occurred in the first few micrograms per deciliter of BPb. Because the authors performed no formal testing of other forms of the dose–response function, the log-linear dose–response relationship for BPb on adult blood pressure should be examined in a pooled data study similar to that used for the IQ study detailed here. If the original data in the studies contributing to the meta-analysis for blood pressure were used in a pooled analysis study, there would be > 50,000 subjects in the study. Such a large study sample would allow testing other nonlinear forms of the dose–response relationship for lead and blood pressure against the log-linear form. Any nonlinear dose–response relationship of the same general form as the log-linear function for blood pressure would have economic and public health repercussions similar to those discussed above for IQ. Over the U.S. population, BPb change measured between 1976 and 1999, Nawrot’s coefficient translates into a 4.1 mm Hg decrease in population systolic blood pressure, a change with significant health and economic benefits. However, most of the health and economic benefits would be realized only by bringing population and occupational exposures well below currently permitted limits.

## Conclusions

Correctly specifying the dose–response or exposure–health relationship in all epidemiologic and toxicologic studies has important scientific, economic, and policy implications. Authors of such studies could take the initiative and apply statistical techniques similar to those discussed in this article to test whether the presented functional form of the dose–response relationship cannot be ruled out by definable statistical criteria. Journal editors and their reviewers can also insist that authors provide such evidence regarding dose–response curves in submitted manuscripts. Adopting these practices will give toxicologists additional clues about mechanisms of effect, will give environmental economists more accurate data for their models, and will give regulators the needed information for evidence-based actions.

If the nonlinear form of the exposure–health effect curve is more appropriate to the data than a linear function, we still have most of our work ahead of us to protect the population from the effects of lead.

## Appendix: Steps for Running *J*-test

Selection between two non-nested models

**Linear Model**


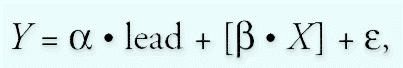


, where [β· *X* ] is the vector of all other variables in the model.

Generate the prediction of linear lead = Πlin.

**Logarithmic Model**


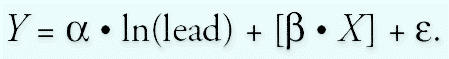


Generate the prediction of natural log lead = Πlog.

**The** **J-test:**


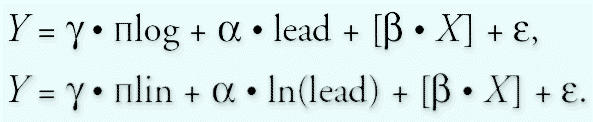


Then test the probability of the predicted lead term and the original lead term in the two artificial regressions above.

## Figures and Tables

**Table 1:** Lead coefficients for IQ as a function of model.^a^

Variable	Coefficient	95% CI	*p*-Value
Linear lead model^b^	−0.18	−0.26 to −0.10	< 0.0005
Natural-log lead model^c^	−2.70	−3.74 to −1.66	< 0.0005
Quadratic lead model	0.005	0.001 to 0.009	0.020
Quadratic-log lead model	−0.25	−0.76 to 0.26	0.258
Ln(lead) with linear lead prediction (*J*-test) model	−2.47	−4.30 to −0.63	0.009

a Control variables for all models were HOME, birth weight, maternal IQ, maternal education, and site identification.

b Model with linear lead specification.

c Model with natural-log lead specification.

**Table 2:** GAM results for IQ.

Variable	df^a^	Gain	Probability of gain
HOME	2	2.621	0.106
Birth weight	2	2.587	0.108
Maternal IQ	2	0.596	0.440
Maternal education	2	0.961	0.327
Linear lead	2	7.467	0.006

df, degrees of freedom. Dichotomous variables (sites) not shown: spline fit of linear lead specification and all independent variables with two degree of freedom splines. Total gain (nonlinearity χ^2^) = 14.232 (5.003 df), p = 0.0142.

a Approximate.

**Table 3:** Spline fit of natural-log lead specification and all independent variables with two degree of freedom splines.

Variable	df^a^	Gain	Probability of gain
HOME	2	2.646	0.104
Birth weight	2	2.515	0.113
Maternal IQ	2	0.603	0.438
Maternal education	2	0.690	0.406
Natural-log lead	2	1.438	0.230

df, degrees of freedom. Total gain (nonlinearity χ^2^) = 7.894 (5.005 df), p = 0.1626.

a Approximate.

**Table 4:** Spline fit of all independent variables with two degree of freedom splines with original natural-log lead variable modeled as is.

Variable	df^a^	Linear coefficient	Gain	Probability of gain
HOME	2	4.51	2.740	0.098
Birth weight	2	1.48	2.523	0.112
Maternal IQ	2	4.91	0.609	0.436
Maternal education	2	1.15	0.642	0.424
Natural-log lead	1	−2.62	—	—

Abbreviations: —, not applicable (natural log lead was not modeled as a spline function; thus, there is no gain or probability of gain); df, degrees of freedom. Total gain (nonlinearity χ2) = 6.514 (4.006 df), p = 0.1644.

a Approximate.

**Table 5:** Economic savings (year 2000 dollars) per cohort estimated from the Grosse et al. (2002) IQ model according to dose–response specification.

Study	Benefit/cohort (billions $)	95% CI^a^
Grosse et al. (2002) linear lead	213.83	147.27–280.39
Pooled analysis, linear lead	148.58	82.18–215.82
Pooled analysis, natural-log lead	318.98	196.30–441.67

a CIs cannot be used to compare linear and log lead specifications because the linear specification is incorrect and the 95% CI calculated from it suffers from uncorrected residual heteroskedasticity.

## References

[b1-ehp0113-001190] Baghurst PA, McMichael AJ, Wigg NR, Vimpani GV, Robertson EF, Roberts RJ (1992). Environmental exposure to lead and children’s intelligence at the age of seven years. The Port Pirie Cohort Study. N Engl J Med.

[b2-ehp0113-001190] Becher H (1992). The concept of residual confounding in regression models and some applications. Stat Med.

[b3-ehp0113-001190] Bellinger DC, Stiles KM, Needleman HL (1992). Low-level lead exposure, intelligence and academic achievement: a long-term follow-up study. Pediatrics.

[b4-ehp0113-001190] Benedetti A, Abrahamowicz M (2004). Using generalized additive models to reduce residual confounding. Stat Med.

[b5-ehp0113-001190] Caldwell BM Bradley R 1984. Home Observation for Measurement of the Environment. Little Rock, AK:University of Arkansas.

[b6-ehp0113-001190] Canfield RL, Henderson CR, Cory-Slechta DA, Cox C, Jusko TA, Lanphear BP (2003). Intellectual impairment in children with blood lead concentrations below 10 microg per deciliter. N Engl J Med.

[b7-ehp0113-001190] CDC 1991. Preventing Lead Poisoning in Young Children. Atlanta, GA:Centers for Disease Control and Prevention.

[b8-ehp0113-001190] Davidson R, MacKinnon J (1981). Several tests for model specification in the presence of alternative hypotheses. Econometrica.

[b9-ehp0113-001190] Dietrich KN, Berger OG, Succop PA, Hammond PB, Bornschein RL (1993). The developmental consequences of low to moderate prenatal and postnatal lead exposure: intellectual attainment in the Cincinnati Lead Study Cohort following school entry. Neurotoxicol Teratol.

[b10-ehp0113-001190] Ernhart CB, Morrow-Tlucak M, Wolf AW, Super D, Drotar D (1989). Low level lead exposure in the prenatal and early preschool periods: intelligence prior to school entry. Neurotoxicol Teratol.

[b11-ehp0113-001190] Grosse SD, Matte TD, Schwartz J, Jackson RJ (2002). Economic gains resulting from the reduction in children’s exposure to lead in the United States. Environ Health Perspect.

[b12-ehp0113-001190] Hastie TJ Tibshirani R 1990. Generalized Additive Models. New York:Chapman & Hall/CRC.

[b13-ehp0113-001190] Lanphear BP, Hornung R, Khoury J, Yolton K, Baghurst P, Bellinger DC (2005). Low-level environmental lead exposure and children’s intellectual function: an international pooled analysis. Environ Health Perspect.

[b14-ehp0113-001190] Mielke HW, Anderson JC, Berry KJ, Mielke PW, Chaney RL, Leech M (1983). Lead concentrations in inner-city soils as a factor in the child lead problem. Am J Public Health.

[b15-ehp0113-001190] Mielke HW, Dugas D, Mielke PW, Smith KS, Gonzales CR (1997). Associations between soil lead and childhood blood lead in urban New Orleans and rural Lafourche Parish of Louisiana. Environ Health Perspect.

[b16-ehp0113-001190] Mielke HW, Gonzales CR, Smith MK, Mielke PW (1999). The urban environment and children’s health: soils as an integrator of lead, zinc, and cadmium in New Orleans, Louisiana, U.S.A. Environ Res.

[b17-ehp0113-001190] Nawrot TS, Thijs L, Den Hond EM, Roels HA, Staessen JA (2002). An epidemiological re-appraisal of the association between blood pressure and blood lead: a meta-analysis. J Hum Hypertens.

[b18-ehp0113-001190] Occupational Safety and Health Administration 1979. Lead. 29CFR1910.1025.

[b19-ehp0113-001190] Pacala SW, Bulte E, List JA, Levin SA (2003). False alarm over environmental false alarms. Science.

[b20-ehp0113-001190] Ramsey JB (1969). Tests for specification errors in classical linear least squares regression analysis. J R Stat Soc Ser B.

[b21-ehp0113-001190] Schnaas L, Rothenberg SJ, Perroni E, Martinez S, Hernandez C, Hernandez RM (2000). Temporal pattern in the effect of postnatal blood lead level on intellectual development of young children. Neurotoxicol Teratol.

[b22-ehp0113-001190] Schwartz J (1994). Low-level lead exposure and children’s IQ: a meta-analysis and search for a threshold. Environ Res.

[b23-ehp0113-001190] Wasserman GA, Liu X, Lolacono NJ, Factor-Litvak P, Kline JK, Popovac D (1997). Lead exposure and intelligence in 7-year-old children: the Yugoslavia Prospective Study. Environ Health Perspect.

[b24-ehp0113-001190] Wechsler D 1967. Manual for Wechsler Preeschool and Primary Scale of Intelligence. San Antonio, TX:The Psychological Corporation.

[b25-ehp0113-001190] Wechsler D 1974. Manual for Wechsler Intelligence Scale for Children—Revised. San Antonio, TX:The Psychological Corporation.

[b26-ehp0113-001190] Wechsler D 1981. WISC-R-Español. Escala de intelligencia revisiada para el nivel escolar. Mexico City:El Manual Moderno, SA.

[b27-ehp0113-001190] Wechsler D 1991. Manual for Wechsler Intelligence Scale for Children. 3rd ed. San Antonio, TX:The Psychological Corporation.

